# Appropriateness of Hospital Admission for Emergency Department Patients with Bronchiolitis: Secondary Analysis

**DOI:** 10.2196/10498

**Published:** 2018-11-05

**Authors:** Gang Luo, Michael D Johnson, Flory L Nkoy, Shan He, Bryan L Stone

**Affiliations:** 1 Department of Biomedical Informatics and Medical Education University of Washington Seattle, WA United States; 2 Department of Pediatrics University of Utah Salt Lake City, UT United States; 3 Homer Warner Research Center Intermountain Healthcare Murray, UT United States

**Keywords:** appropriate hospital admission, bronchiolitis, emergency department, operational definition

## Abstract

**Background:**

Bronchiolitis is the leading cause of hospitalization in children under 2 years of age. Each year in the United States, bronchiolitis results in 287,000 emergency department visits, 32%-40% of which end in hospitalization. Frequently, emergency department disposition decisions (to discharge or hospitalize) are made subjectively because of the lack of evidence and objective criteria for bronchiolitis management, leading to significant practice variation, wasted health care use, and suboptimal outcomes. At present, no operational definition of appropriate hospital admission for emergency department patients with bronchiolitis exists. Yet, such a definition is essential for assessing care quality and building a predictive model to guide and standardize disposition decisions. Our prior work provided a framework of such a definition using 2 concepts, one on safe versus unsafe discharge and another on necessary versus unnecessary hospitalization.

**Objective:**

The goal of this study was to determine the 2 threshold values used in the 2 concepts, with 1 value per concept.

**Methods:**

Using Intermountain Healthcare data from 2005-2014, we examined distributions of several relevant attributes of emergency department visits by children under 2 years of age for bronchiolitis. Via a data-driven approach, we determined the 2 threshold values.

**Results:**

We completed the first operational definition of appropriate hospital admission for emergency department patients with bronchiolitis. Appropriate hospital admissions include actual admissions with exposure to major medical interventions for more than 6 hours, as well as actual emergency department discharges, followed by an emergency department return within 12 hours ending in admission for bronchiolitis. Based on the definition, 0.96% (221/23,125) of the emergency department discharges were deemed unsafe. Moreover, 14.36% (432/3008) of the hospital admissions from the emergency department were deemed unnecessary.

**Conclusions:**

Our operational definition can define the prediction target for building a predictive model to guide and improve emergency department disposition decisions for bronchiolitis in the future.

## Introduction

Bronchiolitis is the inflammation of the bronchioles, the smallest air passages in the lungs, mainly seen in children under 2 years of age in response to viral respiratory infection. More than one-third of the children are diagnosed with bronchiolitis before 2 years of age [1]. Bronchiolitis is the leading cause of hospitalization in children under 2 years of age, and it is responsible for 16% of hospitalizations in this age group [2-5]. Each year in the United States, bronchiolitis incurs about 287,000 emergency department (ED) visits [6], 128,000 hospitalizations [2], and US $1.73 billion of total inpatient cost (2009) [2].

Around 32%-40% of the ED visits for bronchiolitis end in hospitalization [7-9]. As acknowledged in the current clinical guidelines for bronchiolitis [10,11], ED disposition decisions (to discharge or hospitalize) are often made subjectively because of the lack of evidence and objective criteria for bronchiolitis management [4,12]. This causes large practice variation [3,12-23], wasted health care use, increased iatrogenic risk, and suboptimal outcomes due to unnecessary admissions and unsafe discharges [15,21,24]. About 10% of the infants with bronchiolitis experience adverse events during hospitalization [25]. At present, no operational definition of appropriate hospital admission for ED patients with bronchiolitis exists [26]. Yet, such an operational definition is essential for assessing ED care quality and building a predictive model to guide and standardize disposition decisions [26].

**Figure 1:**
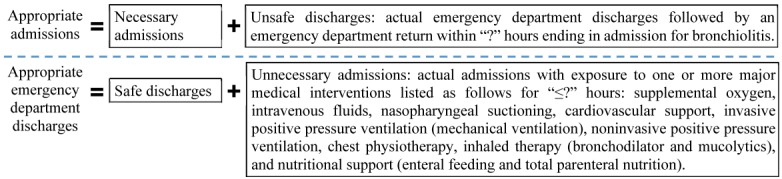
Definition framework of appropriate admission. ?: threshold value.

Our prior work [26] has provided a framework of such an operational definition using 2 concepts: one on safe versus unsafe discharge and another on necessary versus unnecessary hospitalization (Figure 1). Each concept uses a threshold value to be determined. Appropriate admissions include both necessary admissions (actual admissions that are necessary) and unsafe discharges. Appropriate ED discharges include both safe discharges and unnecessary admissions. This study aims to determine the 2 threshold values in a data-driven way, to complete the first operational definition of appropriate hospital admission for ED patients with bronchiolitis, and to report the corresponding percentages of unnecessary admissions and unsafe discharges.

## Methods

### Study Design and Ethics Approval

In this study, we performed secondary analysis of retrospective data. The Institutional Review Boards of the University of Washington Medicine, University of Utah, and Intermountain Healthcare reviewed and approved this study and waived the need for informed consent for all patients.

### Patient Population

Our patient cohort included children under 2 years of age who had ED encounters at 22 Intermountain Healthcare hospitals for bronchiolitis in 2005-2014. Intermountain Healthcare is the largest health care system in Utah, with 185 clinics and 22 hospitals providing ~85% of pediatric care available in Utah [27]. We used an approach similar to that used by Flaherman et al [28-30] to identify as many ED encounters for bronchiolitis as possible.

Several International Classification of Diseases, 9^th^ Revision, Clinical Modification (ICD-9-CM) discharge diagnosis codes, rather than only the discharge diagnosis code of bronchiolitis, can be possibly assigned to an ED encounter for bronchiolitis. Using methods used in prior studies [28-30], we included patients with an ED or hospital ICD-9-CM primary discharge diagnosis of bronchiolitis or bronchitis (466.x), viral pneumonia (480.x), adenoviral infection (079.0), rhinovirus infection (079.3), respiratory infection due to influenza (487.0, 487.1), respiratory syncytial virus (079.6), H1N1 influenza (488.1, 488.11, 488.12), influenza due to identified avian influenza virus (488, 488.0, 488.01, 488.02), or influenza due to novel influenza A (488.81, 488.82).

We also included all patients with any of the above as a nonprimary diagnosis, as long as the ICD-9-CM primary diagnosis was any of the following: apnea (786.03), shortness of breath (786.05), tachypnea (786.06), wheezing (786.07), other respiratory abnormalities (786.09), cough (786.2), fever (780.60, 780.61), acute nasopharyngitis (460), acute upper respiratory infections (465.x), other specified viral infection (079.89), urinary tract infection (599.0), pneumonia unspecified organism (486), unspecified viral infection (079.99), volume depletion (276.5x), or respiratory failure (518.81, 518.82).

### Dataset

We extracted a clinical and administrative data set from Intermountain Healthcare’s enterprise data warehouse. The data set included ED visit and hospitalization information of our patient cohort.

### Data Analysis

To determine the threshold value used for defining unsafe discharges (Figure 1), we examined the length distribution of the interval between an ED discharge and a return ED visit within 2 weeks ending in hospitalization for bronchiolitis [31,32]. In children under 2 years of age, bronchiolitis lasting longer than 2 weeks tends to result from new infection with a differing virus strain instead of persistent infection by the same virus strain [33].

To determine the threshold value used for defining unnecessary admissions (Figure 1), we examined the patients who were hospitalized for ≤12 hours and discharged with no readmission for bronchiolitis within 2 weeks. These patients tended to have been admitted unnecessarily. We used their median duration of using major medical interventions as a conservative threshold for using major medical interventions in all admissions. As shown in Figure 1, major medical interventions include supplemental oxygen, intravenous fluids, nasopharyngeal suctioning, cardiovascular support, invasive positive pressure ventilation (mechanical ventilation), noninvasive positive pressure ventilation, chest physiotherapy, inhaled therapy (bronchodilator and mucolytics), and nutritional support (enteral feeding and total parenteral nutrition) [26]. Every hospital admission with exposure to major medical interventions for no longer than the threshold was deemed unnecessary. During 2005-2012, Intermountain Healthcare iteratively modified its internal guidelines for bronchiolitis management in the ED and hospital several times, with an associated change in the distribution of the duration of using major medical interventions. After the beginning of 2013, significant changes in internal guidelines did not occur. Duration of using major medical interventions became stabilized. To compute the threshold value, we used 2013-2014 data with a stable distribution of duration of using major medical interventions. Both durations of hospitalization and using major medical interventions included only time in the hospital after the patient left the ED.

## Results

Table 1 shows the demographic and clinical characteristics of our patient cohort: children under 2 years of age who had ED encounters for bronchiolitis. About 38.20% (14,292/37,417) of the ED visits for bronchiolitis ended in hospitalization.

**Table 1:** Demographic and clinical characteristics of our patient cohort.

Characteristic		Emergency department visits (n=37,417)	Emergency department discharges (n=23,125)	Emergency department visits ending in hospitalization (n=14,292)
**Age, n (%)**				
	<2 months	4769 (12.75)	1646 (7.12)	3123 (21.85)
	2 to <12 months	22,101 (59.07)	14,569 (63.00)	7532 (52.70)
	12-24 months	10,547 (28.19)	6910 (29.88)	3637 (25.45)
**Gender, n (%)**				
	Male	21,536 (57.56)	13,399 (57.94)	8137 (56.93)
	Female	15,881 (42.44)	9733 (42.06)	6155 (43.07)
**Race, n (%)**				
	American Indian or Alaska native	458 (1.22)	295 (1.28)	163 (1.14)
	Asian	395 (1.06)	222 (0.96)	173 (1.21)
	Black or African American	1017 (2.72)	664 (2.87)	353 (2.47)
	Native Hawaiian or other Pacific islander	2209 (5.90)	1243 (5.38)	966 (6.76)
	White	28,510 (76.20)	17,660 (76.37)	10,850 (75.92)
	Unknown or not reported	4828 (12.90)	3041 (13.15)	1787 (12.50)
**Ethnicity, n (%)**				
	Hispanic	9011 (24.08)	5975 (25.84)	3036 (21.24)
	Non-Hispanic	18,823 (50.31)	11,278 (48.77)	7545 (52.79)
	Unknown or not reported	9583 (25.61)	5872 (25.39)	3711 (25.97)
**Insurance, n (%)**				
	Private	22,162 (59.23)	13,052 (56.44)	9110 (63.74)
	Public	13,448 (35.94)	8729 (37.75)	4719 (33.02)
	Self-paid or charity	1807 (4.82)	1344 (5.81)	463 (3.24)
Asthma, n (%)		2246 (6.00)	883 (3.82)	1363 (9.54)
Chronic complex condition [34], n (%)		2040 (5.45)	365 (1.58)	1675 (11.72)

**Figure 2:**
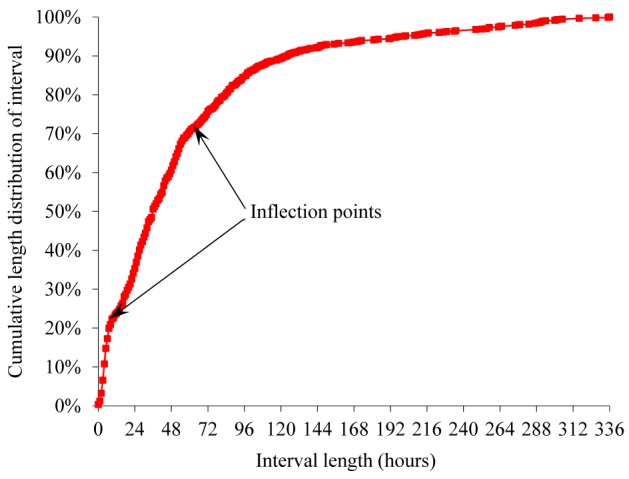
Cumulative length distribution of interval between emergency department discharge and return visit within 2 weeks ending in hospitalization for bronchiolitis.

**Figure 3:**
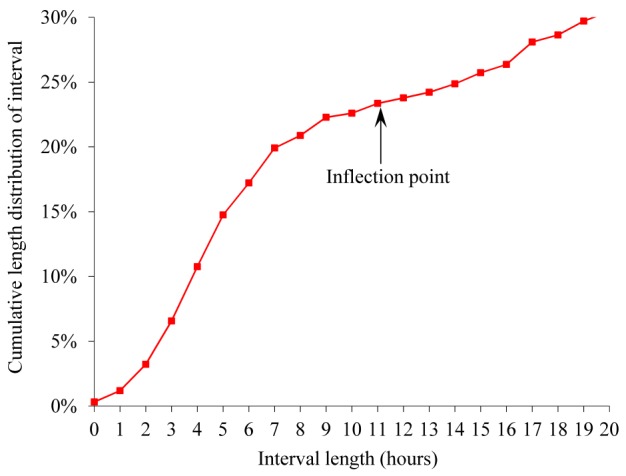
Cumulative length distribution of interval between emergency department discharge and return visit within 2 weeks ending in hospitalization for bronchiolitis, when the interval length is ≤20 hours.

**Figure 4:**
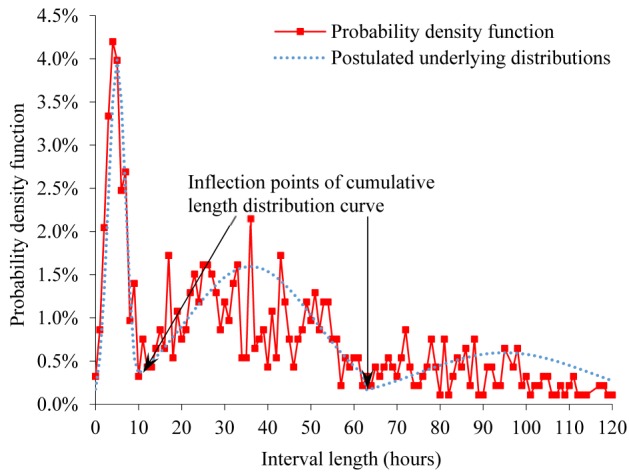
Probability density function of interval between emergency department discharge and return visit within 2 weeks ending in hospitalization for bronchiolitis, when the interval length is ≤120 hours.

Figures 2 and 3 show cumulative length distributions in hours of the interval between an ED discharge and a return ED visit within 2 weeks ending in hospitalization for bronchiolitis. Figure 4 shows probability density function of the interval length. The probability density function was relatively large until the interval length reached the cumulative distribution curve’s inflection point at about 10 to 12 hours and became smaller afterward. The cumulative distribution curve seemed to have 2 inflection points, suggesting 3 underlying distributions. As indicated by the dotted curve in Figure 4, the 3 distributions are postulated to represent an early ED return after an inappropriate ED discharge, natural disease progression in a subgroup of appropriate ED discharges, and an even later ED return due to a new viral infection after an appropriate ED discharge, respectively. When selecting the threshold value for defining unsafe discharges (Figure 1), we wanted our choice to capture the majority of unsafe discharges while avoiding contamination with ED returns not due to unsafe discharges. To help make the choice, we used the probability density function that has a local minimum at the interval length of 10 to 12 hours. We chose 12 hours, which fulfilled our selection criteria. Accordingly, 0.96% (221/23,125) of the ED discharges were followed by an ED return within 12 hours resulting in hospital admission for bronchiolitis and were deemed unsafe ED discharges.

**Figure 5:**
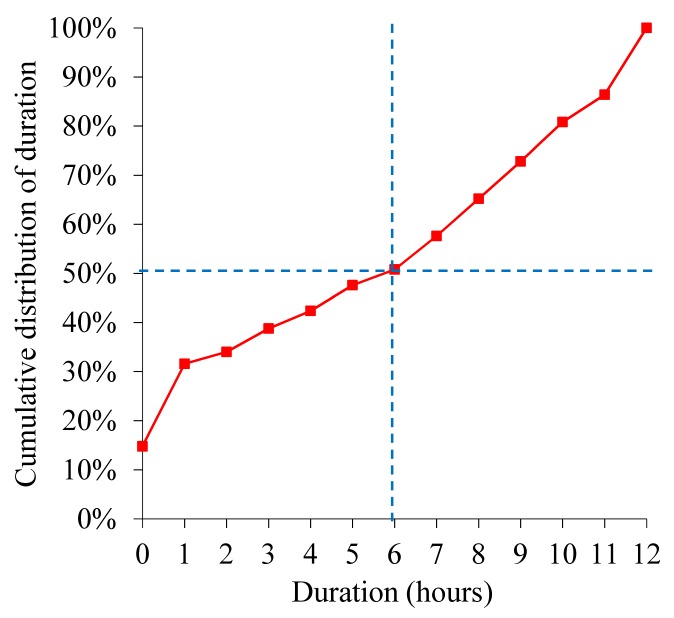
Distribution of duration of using major medical interventions among patients who were hospitalized for ≤12 hours in 2013-2014 and discharged with no readmission for bronchiolitis within 2 weeks.

In 2013-2014, no major medical intervention was applied in 6.45% (194/3008) of the cases of hospitalization from the ED for bronchiolitis. In another 7.91% (238/3008) of the cases, 1 or more major medical interventions were applied, but the duration of using them was ≤6 hours. Among the patients hospitalized in 2013-2014, 8.31% (250/3008) were hospitalized for ≤12 hours and discharged with no readmission for bronchiolitis within 2 weeks. Figure 5 shows the distribution of duration of using major medical interventions in these patients. Median duration of using major medical interventions was 6 hours, which we used as the threshold value for defining unnecessary admissions (Figure 1). Accordingly, 14.36% (432/3008) of the hospital admissions from the ED in 2013-2014 incurred exposure to major medical interventions for no longer than this threshold and were deemed unnecessary.

By filling in the 2 threshold values in our definition framework (Figure 1) [26], we completed the first operational definition of appropriate hospital admission for ED patients with bronchiolitis. Appropriate hospital admissions include actual admissions with exposure to major medical interventions for more than 6 hours, as well as actual ED discharges followed by ED return within 12 hours ending in admission for bronchiolitis. Putting unsafe ED discharges and unnecessary admissions together, 6.08% of the ED disposition decisions for bronchiolitis were deemed inappropriate.

## Discussion

### Principal Findings

We completed the first operational definition of appropriate hospital admission for ED patients with bronchiolitis. The definition uses 2 concepts, one on safe versus unsafe discharge and another on necessary versus unnecessary hospitalization. Based on the definition, we found that many ED disposition decisions for bronchiolitis were deemed inappropriate. Our findings highlight opportunities for improving ED disposition decisions and the need to build a model to predict appropriate admission. The model could become the foundation of a decision support tool to help make appropriate ED disposition decisions for bronchiolitis, improve bronchiolitis outcomes, and cut health care costs [26]. Although the model could be built without using the ED physician’s initial, tentative disposition decision as an input variable, the model would likely be more accurate if this variable is included. In either case, physicians can use the model’s output to give a second thought on their initial, tentative disposition decision.

### Comparison With Prior Work

Some aspects of our findings are similar to those in previous studies. In our data set, about 38.20% (14,292/37,417) of the ED visits for bronchiolitis ended in hospitalization. This percentage is close to the corresponding percentages (32%-40%) reported in the literature [7-9]. For 30 EDs in 15 US states, Norwood et al [35] have presented the length distribution of interval between an ED discharge and a return ED or clinic visit within 2 weeks for bronchiolitis. That distribution is similar to the one we have shown in Figure 2, which presents the length distribution of interval between an ED discharge and a return ED visit within 2 weeks ending in hospitalization for bronchiolitis.

Some of our findings are different from those in previous studies. In our dataset, 14.36% (432/3008) of the hospital admissions from the ED in 2013-2014 were deemed unnecessary. This percentage is smaller than the corresponding percentages (20%-29%) suggested in the literature [36,37]. Intermountain Healthcare has multiple collaborative partnerships among its EDs and hospitals to ensure that pediatric specialty care is co-ordinated and not focused just in a tertiary pediatric hospital. Several quality improvement projects for bronchiolitis management were completed during 2005-2012, impacting the ED and hospital care of children in multiple hospitals within Intermountain Healthcare. The average quality of ED disposition decisions for bronchiolitis made at Intermountain Healthcare could be higher than that of ED disposition decisions for bronchiolitis made at some other health care systems, particularly if those health care systems employ few pediatricians in their EDs.

### Limitations

This study has several limitations. One limitation is that the study used data from a single health care system, Intermountain Healthcare, and our results may not be generalized to other health care systems. Notably, most Intermountain Healthcare hospitals are at a high elevation (more than 4000 feet above sea level). This may result in increased incidence of hypoxia. About 46% of the patients hospitalized with bronchiolitis at Intermountain Healthcare are discharged on home oxygen for outpatient management. Protocols are in place to facilitate brief hospitalizations if oxygen is the only intervention a patient needs in the hospital [30]. In the future, it would be desirable to use data from other health care systems to validate our operational definition of appropriate hospital admission for ED patients with bronchiolitis. As indicated by similarities between our findings and those in previous studies, we do not expect such validation to significantly change our results. Intermountain Healthcare is a large health care system with EDs at 22 heterogeneous hospitals spread over a large geographic area, ranging from community metropolitan and rural hospitals attended by general practitioners and family doctors with constrained pediatric resources to tertiary care children’s and general hospitals in urban areas attended by subspecialists [26]. Each hospital has a different patient population, geographic location, staff composition, scope of services, and cultural background. This variation provides a realistic situation for finding factors generalizable to other hospitals across the United States.

Another limitation of this study is that Intermountain Healthcare does not have complete clinical and administrative data on all of its patients although it is an integrated health care system. Within 2 weeks of a visit to an Intermountain Healthcare ED for bronchiolitis, a patient could use a non-Intermountain Healthcare hospital for bronchiolitis again. If this occurred, our data set would miss the information on health care use that occurred at a non-Intermountain Healthcare hospital. Including data from non-Intermountain Healthcare hospitals may lead to different results. Nevertheless, we do not expect this to greatly change the accuracy of our results. Intermountain Healthcare provides ~85% of the pediatric care available in Utah [27]. Thus, our dataset is reasonably, although not 100%, complete in terms of capturing bronchiolitis patients’ use of hospitals at Utah.

A third limitation is that this study does not consider factors, such as preference of the patient’s parents, patient transportation availability, and time of day, while defining appropriate hospital admission. Many of these factors are often undocumented in patient records. For some hospital admissions from the ED that were deemed unnecessary based on our operational definition of appropriate hospital admission, the original admission decisions could be made due to these factors.

### Conclusions

We provided the first operational definition of appropriate hospital admission for ED patients with bronchiolitis. Our operational definition can define the prediction target for building a predictive model in the future with the goal of standardizing and improving ED disposition decisions for bronchiolitis.

## Abbreviations

ED: emergency department
ICD-9-CM: International Classification of Diseases, Ninth Revision, Clinical Modification
